# Special Issue: Characterization of Extracellular Vesicles in Disease

**DOI:** 10.3390/ijms26167880

**Published:** 2025-08-15

**Authors:** Verena Tretter

**Affiliations:** Clinical Division of General Anesthesia and Intensive Care Medicine, Medical University of Vienna, 1090 Vienna, Austria; verena.tretter@meduniwien.ac.at

Extracellular vesicles as components of the cellular secretome are important mediators of inter-cellular communication via auto-, para-, and endocrine routes [1]. They are generated by plasma membrane blebbing or originate from the internal endosomal system and are actively or passively loaded with cytosolic molecules such as proteins, metabolites, mRNAs, miRNAs and more [2]. As such, they are highly heterogenous in size and composition, but contain signature molecules of their parent cells, that allow insight into their physiological state and eventually pathological change. As extracellular vesicles are found in all body fluids, sample collection is easy. Some of their surface proteins are characteristic makers of their tissue-of origin, therefore their potential as biomarker is increasingly exploited for therapeutic decision making. Extracellular vesicles are not only a kind of cell waste system in the body, but they are taken up by target cells by several different mechanisms, where their cargo elicits biological effects. Extracellular vesicles from stem cells have been shown to transfer their regenerative potential to injured tissues [3]. Progress in molecular biology has provided new techniques to further engineer the surface and cargo of the vesicles in order to improve therapeutic effects, suppress negative side-effects and to even use the vesicles as drug carriers for targeted therapy [4].

All applications of extracellular vesicle research are confronted with the major challenge of vesicle heterogeneity. This comprises size, intra-lumenar cargo and membrane properties with regard to lipid composition, glycosylation patterns, transmembrane and peripheral proteins. The biological heterogeneity also connotes technical difficulties with the isolation of pure vesicles preparations, therefore a whole consortia of extra vesicle experts and researchers aim to standardize purification and analysis protocols, which are updated on a regular basis as techniques and instrumentation are improved and progress is made.

This Special Issue comprises six articles (four Original Works and two Reviews) that reveal different examples of analyzing extracellular vesicles as indicators (and probably also mediators) of pathological conditions and to use them as a therapeutic strategy with the help of modern engineering techniques to improve the beneficial outcome.

The first study by Zipperle et al. [5] describes an approach to use circulating large extracellular vesicles (lEV) subsets in the decision making of the dose of unfractionated heparin (UFH) for anti-coagulation in veno-venous extracorporeal membrane oxygenation (ECMO). ECMO is an intensive care method of cardiorespiratory support in cases of heart/respiratory failure, where the blood is diverted from the vascular system to an extracorporeal loop containing an oxygenator before being reinfused into the circulation. While the vascular endothelium has natural means of preventing spontaneous thrombosis, this is not the case when the blood comes into contact with tubings, pumps and oxygenators. Therefore, a personalized anticoagulation regime is necessary in ECMO patients to prevent thrombosis on one side and life-threatening bleeding on the other side. Closely linked to coagulation is also the inflammation issue in ECMO patients, which is due to shear stress, contact with artificial surfaces and activation of thrombocytes. lEVs (microvesicles from ectosomal membrane blebbing) have gained special recognition in severely ill patients, as they frequently expose pro-coagulant surface phosphatidylserine or tissue factor and promote multi-organ dysfunction. The authors show in their post hoc analysis of a multicenter randomized controlled trial that low doses of UFH (<15 IU/kg(h) induce the abundance of a higher number of endothelial Annexin^+^ (=phosphatidylserine^+^, PS^+^) lEVs, while higher doses of UFH increase the amounts of lEVs derived from red blood cells in addition to more Annexin^+^ lEVs carrying mitochondria. The optimal choice of anti-coagulant type and dose is complex and a matter of ongoing debate with the aim of personalized intervention. Current monitoring tests use aPTT (Activated Partial Thromboplastin Time), ACT (Activated Clotting Time), Anti-Xa-Assay or Viscoelastic Assays. Analysis of extracellular vesicles can give insight in several fashions: they transduce pro-inflammatory and pro-thrombotic information throughout the circulation to other parts of the body. PS^+^ lEV are indicators of cell death and provide an additional docking site for coagulation factor complexes. Their occasional content of mitochondria has an impact on inflammation.

In order to better understand the role of extracellular vesicles in disease, the analysis of their proteome is of major interest [6]. Several different approaches are available to qualitatively and quantitatively assess the abundance of proteins on the vesicle surface or as part of their cargo. Distinct proteins on the vesicle surface can be tagged with fluorescent antibodies and quantified by flow cytometry analysis. The whole proteome of the vesicle is determined by different variants of mass-spectrometry analysis, whereby a distinction of surface proteins can be achieved by additional labeling, for instance by biotinylation. Label-free approaches are relatively simple and especially cost-efficient; however, more accurate quantification can be achieved by chemical or metabolic labeling, such as iTRAQ (isobaric tags for relative and absolute quantitation), TMT (Tandem Mass Tag) multiplex and SILAC (Stable Isotope Labeling with Amino acids in Cell culture). SILAC can be adapted to human samples such as plasma by using a super-SILAC spike-in mix generated from several metabolically labeled cell lines that serve as an internal standard [7]. Depending on the goal of the study, data-dependent acquisition (DDA) or data-independent acquisition (DIA) is chosen. DDA is suitable for small-scale studies and provides high sensitivity and accuracy, for instance, in the detection of post-translational modifications, while DIA excels in larger-scale complex studies providing high coverage (including low-abundance proteins) and quantitative accuracy.

Resch et al. [8] use a SILAC/DDA approach in a small study to elucidate distinct protein signatures in plasma-derived extracellular vesicles from patients undergoing partial hepatectomy (PHx) after diagnosis with hepatocellular carcinoma (HCC). One group of patients underwent a special two-stage surgical method, ALPPS (Associating Liver Partition and Portal vein ligation for Staged hepatectomy). In ALPPS, the healthy part of the liver is first separated from the cancerous tissue and then is allowed to grow by portal vein ligation of the latter, before the cancerous part is removed in a second surgery. Prognostic markers for a potential subsequent post-hepatectomy liver failure (PHLF) are scarce and extracellular vesicles in the plasma could give insight into preexisting conditions that enhance the likelihood of PHLF. Despite the small sample size and limitations due to contamination of plasma vesicles with co-purifying blood proteins during ultracentrifugation, the authors found quantitatively significant diverse protein content in the three treatment groups that indicate different cellular processes from enrichment analyses.

Cell culture experiments with primary cells from human tissue are frequently the first approach in mechanistic studies, as these systems reduce the complexity of a multicellular organ composed of different cell types, but are still relevant to the in vivo situation, as freshly isolated primary cells still maintain most of their native properties. The following two studies by Yeung et al. and Schaubmayr et al. both use similar experimental set-ups of 2D cell cultures, the isolation of extracellular vesicles from conditioned cell culture supernatants followed by (TMT^TM^) 10-plex labeling and mass spectrometry.

In order to better understand inter-cellular communication via extracellular vesicles in the human cornea, Yeung et al. [9] isolated and cultured the different corneal cell types epithelial cells, keratocytes, fibroblasts and myofibroblasts. Results show specific protein signatures, which relate well with the biological function of the individual cell-types.

Schaubmayr et al. [10] simulated different oxygen conditions in the pulmonary microvasculature (hypoxia, hyperoxia, normoxia, intermittent hypoxia and hyperoxia) as encountered in states of lung disease in cell culture. They show that oscillating oxygen conditions induce an increase in the release of extracellular vesicles with an increased exposure of phosphatidylserine and tissue factor on the vesicle surface. Tissue factor activity, however, was only increased under intermittent hypoxia, but not intermittent hyperoxia. Subsequent proteome analysis of the vesicle cargo showed that increased amounts of the tissue factor-activating enzymes, protein disulfide isomerase and acid sphingomyelinase, under intermittent hypoxia. This observation implicates a specific loading into vesicles under this condition prior to release from the parent cell. Under intermittent hyperoxia, tissue factor might remain encrypted on the surface of extracellular vesicles. The whole cargo proteome of extracellular vesicles was consistent with signaling typical for cell responses under these oxygen conditions. Therefore, extracellular vesicles communicate the situation in the lung microvasculature via the circulation to other parts and organs of the body, and might, in addition to soluble factors such as cytokines, contribute to biotrauma.

The final two papers in this Special Issue review the current state of research of the role of extracellular vesicles in a disease (acute kidney injury, AKI) (Norgard and Svenningsen [11]), and technical approaches of vesicle engineering for therapeutic use (Ziegler and Tian [12]).

Acute kidney injury is frequently associated with traits of ischemia/reperfusion inducing release of reactive oxygen species (ROS), activation of HIF and NF-KB, induction of inflammation and apoptosis, which altogether can ultimately lead to organ dysfunction. Some of these processes most likely are co-mediated by extracellular vesicles that can aggravate or ameliorate these processes with their cargo. They are also means of communication between different kidney cell types, which need to be understood in more detail, but are frequently hampered by the technical limitations of sampling and analysis methodology. Kidney glomerular filtration represents a barrier withholding plasma vesicles. Extracellular vesicles in the urine are mainly derived from the urogenital system and kidney epithelium and their abundance increases with hypoxic conditions implicating their potential use as biomarkers for kidney function.

Extracellular vesicles are not only prognostic and diagnostic biomarkers, but can be used for therapeutic applications. Extracellular vesicles in the secretome of mesenchymal stem cells have been attributed the main bio-active regenerative effects in stem cell therapy and, by themselves, are regarded as being safer than cell therapy. In order to further boost their therapeutic potency and to reduce putative negative side-effects, extracellular vesicles can be engineered. Engineering comprises alterations in vesicle cargo and the addition of targeting cues, which support their accumulation in the target organ or cell type. These techniques have been successfully applied to treat cancers, neurological and cardiovascular disease. Ziegler and Tian present a comprehensive overview of different studies that use engineered extracellular vesicles to treat a diverse spectrum of diseases, discuss encountered pitfalls and provide an outlook on possible future developments in this field.

## References

[1] van Niel G., Carter D.R.F., Clayton A., Lambert D.W., Raposo G., Vader P. (2022). Challenges and directions in studying cell-cell communication by extracellular vesicles. Nat. Rev. Mol. Cell Biol..

[2] Dixson A.C., Dawson T.R., Di Vizio D., Weaver A.M. (2023). Context-specific regulation of extracellular vesicle biogenesis and cargo selection. Nat. Rev. Mol. Cell Biol..

[3] Matsuzaka Y., Yashiro R. (2022). Therapeutic Strategy of Mesenchymal-Stem-Cell-Derived Extracellular Vesicles as Regenerative Medicine. Int. J. Mol. Sci..

[4] Cheng L., Hill A.F. (2022). Therapeutically harnessing extracellular vesicles. Nat. Rev. Drug Discov..

[5] Zipperle J., Vock L., Fritsch G., Grillari J., Osuchowski M.F., Holnthoner W., Schochl H., Halbgebauer R., Huber-Lang M., Hofmann N. (2024). Effect of Unfractionated Heparin Dose on Complement Activation and Selected Extracellular Vesicle Populations during Extracorporeal Membrane Oxygenation. Int. J. Mol. Sci..

[6] Mallia A., Gianazza E., Zoanni B., Brioschi M., Barbieri S.S., Banfi C. (2020). Proteomics of Extracellular Vesicles: Update on Their Composition, Biological Roles and Potential Use as Diagnostic Tools in Atherosclerotic Cardiovascular Diseases. Diagnostics.

[7] Geiger T., Cox J., Ostasiewicz P., Wisniewski J.R., Mann M. (2010). Super-SILAC mix for quantitative proteomics of human tumor tissue. Nat. Methods.

[8] Resch U., Hackl H., Pereyra D., Santol J., Brunnthaler L., Probst J., Jankoschek A.S., Aiad M., Nolte H., Krueger M. (2024). SILAC-Based Characterization of Plasma-Derived Extracellular Vesicles in Patients Undergoing Partial Hepatectomy. Int. J. Mol. Sci..

[9] Yeung V., Boychev N., Kanu L.N., Ng V., Ross A.E., Hutcheon A.E.K., Ciolino J.B. (2024). Proteomic Characterization of Corneal Epithelial and Stromal Cell-Derived Extracellular Vesicles. Int. J. Mol. Sci..

[10] Schaubmayr W., Hochreiter B., Hunyadi-Gulyas E., Riegler L., Schmidt K., Tiboldi A., Moser B., Klein K.U., Krenn K., Scharbert G. (2024). The Proteome of Extracellular Vesicles Released from Pulmonary Microvascular Endothelium Reveals Impact of Oxygen Conditions on Biotrauma. Int. J. Mol. Sci..

[11] Norgard M.O., Svenningsen P. (2023). Acute Kidney Injury by Ischemia/Reperfusion and Extracellular Vesicles. Int. J. Mol. Sci..

[12] Ziegler J.N., Tian C. (2023). Engineered Extracellular Vesicles: Emerging Therapeutic Strategies for Translational Applications. Int. J. Mol. Sci..

